# Interest in Cancer Predisposition Testing and Carrier Screening Offered as Part of Routine Healthcare Among an Ethnically Diverse Sample of Young Women

**DOI:** 10.3389/fgene.2022.866062

**Published:** 2022-04-14

**Authors:** Kimberly A. Kaphingst, Jemar R. Bather, Brianne M. Daly, Daniel Chavez-Yenter, Alexis Vega, Wendy K. Kohlmann

**Affiliations:** ^1^ Department of Communication, University of Utah, Salt Lake City, UT, United States; ^2^ Huntsman Cancer Institute, University of Utah, Salt Lake City, UT, United States; ^3^ Department of Biostatistics, Harvard T.H. Chan School of Public Health, Boston, MA, United States

**Keywords:** population screening, genetic testing, cancer predisposition testing, carrier screening, ethnicity

## Abstract

Sequencing technologies can inform individuals’ risks for multiple conditions, supporting population-level screening approaches. Prior research examining interest in genetic testing has not generally examined the context of population-based approaches offered in routine healthcare or among ethnically diverse populations. Cancer predisposition testing and carrier screening could be offered broadly to women of reproductive age. This study therefore examined interest in these tests when offered as part of routine care, and predictors of interest, among an ethnically diverse sample of women aged 20–35. We conducted an online English-language survey of 450 women; 39% identified as Latina. We examined predictors of interest for two outcomes, interest in testing in the next year and level of interest, in multivariable logistic regression models and stratified analyses by Latina ethnicity. More than half of respondents reported being interested in cancer predisposition testing (55%) and carrier screening (56%) in the next year; this did not differ by ethnicity. About 26% reported being very interested in cancer predisposition testing and 27% in carrier screening. Latina respondents (32%) were more likely to be very interested in cancer predisposition testing than non-Latina respondents (22%; *p* < 0.03). In multivariable models, having higher worry about genetic risks, higher genetic knowledge, and higher perceived importance of genetic information were associated with higher interest across multiple models. Predictors of interest were generally similar by ethnicity. Our findings show substantial interest in both cancer predisposition testing and carrier screening among young women as part of routine healthcare with similar interest between Latina and non-Latina women. Efforts to broadly offer such testing could be important in improving access to genetic information. It will be critical to develop tools to help healthcare providers communicate about genetic testing and to address the needs of those who have less prior knowledge about genetics to support informed decision making.

## 1 Introduction

DNA-based population screening of unaffected individuals has been identified as an important future approach to inform individual disease risks and direct screening and prevention efforts (Murray et al., 2021). Currently, genetic testing is generally targeted based on medical history factors, such as family history and personal history of disease (Murray et al., 2019). However, increasing evidence shows that medical history-based genetic testing approaches do not identify the majority of individuals at increased inherited risk for cancer and heart disease (Abul-Husn et al., 2016; Manickam et al., 2018; Khoury and Dotson, 2021). These gaps in identification, combined with decreasing costs of sequencing technologies, have led to heightened consideration of population screening approaches (Murray et al., 2019; Murray et al., 2021). Tier 1 genomic applications, which are hereditary breast and ovarian cancer, Lynch Syndrome, and familial hypercholesterolemia, have received particular consideration for future implementation of population screening (Khoury et al., 2018; Khoury and Dotson, 2021).

A number of recent commentaries have outlined key questions that need to be addressed prior to launching population screening efforts (Murray et al., 2019; Bean et al., 2021; Khoury and Dotson, 2021; Murray et al., 2021). Although previous research studies have begun to explore population-based testing approaches in defined populations, such as BRCA testing among an Ashkenazi Jewish population (Manchanda et al., 2020a; Manchanda et al., 2020b), limited data exist to inform the implementation of population screening more broadly and the potential impact on health outcomes (French et al., 2018; Phillips et al., 2020). Related data that are available suggest that population screening could have several behavioral benefits, such as increased screening in women at high risk of breast cancer without major adverse emotional effects (French et al., 2018). However, substantial gaps have been identified in data related to how individuals would make decisions related to offers of population screening (French et al., 2018). One important need is to understand individuals’ interest in population screening for various disease outcomes, and the factors that influence their interest. These findings are critical to developing effective approaches to offering population screening and supporting individuals’ informed decision making. The importance of these issues is likely to increase as the public becomes more interested in obtaining their genomic information (Bean et al., 2021).

In considering potential future population screening initiatives, pre-pregnancy may offer a unique opportunity to engage women and their reproductive partners in genetic testing. Pre-pregnancy has been identified as a key window for health promotion activities (Johnson et al., 2006; Barker et al., 2017; American College of Obstetrics and Gynecology, 2019a; van Elten et al., 2019; Hill et al., 2020; Moholdt and Hawley, 2020). While definitions of pre-pregnancy vary (Hill et al., 2020), women who are intending a pregnancy in the future may be particularly interested in various types of genetic information. The Centers for Disease Control and Prevention have identified genetic conditions and family history as specific areas for pre-pregnancy risk assessment (Johnson et al., 2006). Carrier screening is a recommended genetic test to identify couples at risk for conceiving a fetus affected with a serious health condition that can be offered pre-pregnancy (Porter et al., 2018). Currently, the American College of Obstetricians and Gynecologists (ACOG) and American College of Medical Genetics and Genomics (ACMG) recommend that all couples be offered carrier screening for cystic fibrosis and spinal muscular atrophy, and other targeted screening based on ethnicity (Edwards et al., 2015; American College of Obstetrics and Gynecology, 2017a; American College of Obstetrics and Gynecology, 2017b). However, expanded carrier screening, which potentially screens for hundreds of conditions, could be offered more broadly at a population level.

While carrier screening is ideally offered prior to pregnancy, it is often not offered until a pregnancy when results could increase anxiety to a greater extent due to the high likelihood of carrier status for one or more conditions and time for partner results (Grody, 2016). In considering population screening efforts for expanded carrier screening, therefore, some research has examined interest among women, as well as their reproductive partners, in receiving this genetic testing prior to pregnancy (Capalbo et al., 2021). Wide variability in interest and uptake of expanded carrier screening has been observed across available studies (van Steijvoort et al., 2020). A systematic review of 12 published studies found that 32%–76% of respondents were interested in a hypothetical expanded carrier screening test, while actual uptake rates for expanded carrier screening ranged from 8% to 50% (van Steijvoort et al., 2020). While the highest uptake rate was observed in a study with pregnant women (van Steijvoort et al., 2020), another study that compared uptake rates found that 69% of women counseled pre-pregnancy chose to have expanded carrier screening, which was significantly higher than the 35% choosing to have screening during pregnancy (Larsen et al., 2019).

This wide range of interest and uptake observed in different studies with different populations heightens the importance of examining factors affecting interest if expanded carrier screening were offered pre-pregnancy to a broad population. A few studies have examined women’s reasons for choosing to have or declining pre-pregnancy carrier screening. In one survey of the general Dutch population, the primary motivation for receiving expanded carrier screening was to spare a child from a life with a severe hereditary disorder, while lack of a hereditary disorder in the family was identified as a reason to decline screening (Nijmeijer et al., 2019). Another survey identified the desire for reassurance and making informed decisions about future pregnancies as drivers of interest in expanded carrier screening (Rabkina et al., 2021). Interestingly, in one study, women who declined offers of preconception genomic carrier screening did so for logistical issues (e.g., time) rather than the rationale for testing (Gilmore et al., 2017). Limited prior research has examined psychological predictors of interest in preconception carrier screening, although one study in Western Australia found that higher genetic knowledge and more positive attitudes were correlated with screening interest (Ong et al., 2018).

Use of sequencing technologies for expanded carrier screening could allow for informing risks for other health conditions among those receiving genetic testing (Lindor et al., 2017; Machini et al., 2019). Routine gynecology visits may be an ideal time for women to consider both expanded carrier screening and genetic testing for cancer predisposition, as these are both clinical genetic tests that are highly relevant to women of reproductive age. ACOG recommends that assessing for hereditary cancer risk and offering carrier screening are within the roles of obstetrics/gynecology providers (American College of Obstetrics and Gynecology, 2017c; American College of Obstetrics and Gynecology, 2019a; American College of Obstetrics and Gynecology, 2019b), and that familial cancer risk assessment be part of routine gynecological visits (Gavin et al., 2014). Returning multiple types of genetic information may bring substantial communication challenges due to greater information volume and complexity but returning multiple results may also increase the perceived value of genetic testing to individuals (Lindor et al., 2017; Kaphingst et al., 2018; Sapp et al., 2018; Delanne et al., 2019; Horowitz et al., 2019; Bartley et al., 2020). While studies have begun to explore interest in offers of pre-pregnancy genomic carrier screening (Kauffman et al., 2017b; van Steijvoort et al., 2020), research is needed to assess women’s interest in receiving additional genetic tests that would provide information about their own health at the same time and whether predictors of interest are the same between different types of genetic tests. One prior study related to participating in genome sequencing for carrier status showed that a primary motivating factor was to obtain general health information for oneself (Kauffman et al., 2017a). Additional research is needed to examine whether interest in both of these types of genetic tests would be high in a routine clinical setting as well.

Prior related research conducted outside of the pre-pregnancy and carrier status context has shown that patients are often interested in receiving multiple types of genetic information from genome sequencing, including cancer risk information (Kaphingst et al., 2016a; Kaphingst et al., 2018; Delanne et al., 2019; Hoell et al., 2020). Many of these studies have been conducted in the context of genome sequencing research rather than routine clinical contexts, finding high levels of interest in secondary findings related to various health conditions among the general public and patient populations (Kaphingst et al., 2019). Studies have found strong interest in receiving secondary findings among cancer patients, with the strongest interest in actionable findings and those with reproductive significance (Kaphingst et al., 2016a; Kaphingst et al., 2018; Bijlsma et al., 2020). Members of the general public have also perceived genome sequencing results as having high personal utility (Goranitis et al., 2020). A number of different factors affecting interest in various types of sequencing results have been identified (Mighton et al., 2019), including understanding and impact on quality of life (Bollinger et al., 2012; Mighton et al., 2020). Early adopters of genome sequencing have expressed various health-related and non-health-related motivations (Sanderson et al., 2016), and participants in genetic research have highlighted the importance of offers of personal genomic risk information being based on individual preferences (Smit et al., 2020).

Our prior work has examined possible predictors of interest in various types of findings from genome sequencing informed by a model of risk information and processing (Griffin et al., 1999), examining both genetic-related and general health-related predictors. In one study with 1,080 women who had been diagnosed with breast cancer at a young age, we found that the same psychological factors (i.e., higher knowledge about sequencing benefits, greater worry about genetic risks, and stronger orientation toward health information) predicted a high level of interest in learning about six different types of genome sequencing findings, including carrier status (Kaphingst et al., 2018). In other research conducted with primary care patients offered genetic susceptibility testing for multiple health conditions, we found that social influence from family and friends impacted interest in seeking information about genes (Hay et al., 2012). Additional possible predictors of interest in different types of genetic testing are suggested by related theories of how individuals cope with the uncertainty inherent in risk information (Brashers, 2001; Hillen et al., 2017), particularly the importance of examining individuals’ tolerance for uncertainty information (Carleton et al., 2007; Hillen et al., 2017).

Issues of equity must be considered when assessing interest in population screening, as well as predictors of interest, so that these technologies do not further exacerbate health disparities (Institute of Medicine, 2002; Halbert and Harrison, 2018; Pierle and Mahon, 2019; Murray et al., 2021). There has been limited research on the access and use of genetic technologies among diverse patients (Canedo et al., 2019; Kaphingst et al., 2019), particularly with Latinx patients (Canedo et al., 2020; Chavez-yenter et al., 2021a). For example, people from racial and ethnic minority groups are often interested in testing (Kaphingst et al., 2015; Hay et al., 2019; Turbitt et al., 2019), but have lower access to and use of cancer genetic services in the US (Hall and Olopade, 2005; Hall and Olopade, 2006; Fisher et al., 2019), even when cost barriers are minimized (Alford et al., 2011). These disparities have been linked to both individual-level (e.g., lower knowledge) (Singer et al., 2004; Pagan et al., 2009; Kinney et al., 2010; Bloss et al., 2018; Canedo et al., 2019) and system-level factors (e.g., unmet needs for discussion of testing with providers) (Peters et al., 2004; Singer et al., 2004; Jagsi et al., 2015; Kaphingst and Goodman, 2016; Roberts et al., 2019; Southwick et al., 2020). However, these critical issues need to be examined within the context of population screening approaches.

Prior related research has indicated that a broad population sample may be interested in receiving genetic testing for multiple health conditions, including cancer predisposition testing and carrier status, with at least some support for expanded cancer screening offered pre-pregnancy. However, these studies have not generally been conducted in a clinical setting and little is known about individuals’ interest in genetic testing offered as part of routine healthcare. In addition, research examining predictors of interest in different types of genetic testing among racially and ethnically diverse populations is limited. To address these identified research gaps, this study examined interest, and predictors of interest, in population-based carrier screening and cancer predisposition testing offered as part of routine gynecologic care among an ethnically diverse sample of women aged 20–35.

## 2 Methods

### 2.1 Participants

We conducted an online English-language survey in order to investigate these research questions (see Supplemental File). A convenience sample of US adults was recruited by Qualtrics Panel Services in June 2021 to participate in the survey. Because of our focus on genetic testing pre-pregnancy, we recruited respondents who identified as female and were between the ages of 20–35 years. Because of the limited prior data for Latinx individuals related to use of genetic technologies, as described above, and because of the substantial and growing Latinx community in the catchment area for our healthcare system, we also set an a priori threshold of at least 25% of respondents identifying as Latina so that we could examine the effect of ethnicity on interest. The minimum survey sample size was set at 425 respondents in order to examine the effect of ethnicity on interest in genetic testing. Individuals were removed if they did not meet the gender (*n* = 41) or age (*n* = 34) criteria in the pre-screener questions, did not complete the consent acceptance question at the beginning of the survey (*n* = 51), or were below the 6-min speed threshold pre-set for time to complete the survey (*n* = 52). This resulted in a final sample of 450 respondents. The survey was approved as an exempt protocol by the University of Utah Institutional Review Board.

### 2.2 Measures

#### 2.2.1 Interest Outcome Variables

We began the survey with an educational component that described different types of genetic testing and then asked participants a series of questions about their interest in the different types. Because of our prior work showing that predictors of interest may vary depending upon item wording (Guo et al., 2020), we assessed interest in genetic testing with two different item formats. Five items assessed respondents’ level of interest in genetic testing for cancer predisposition testing (i.e., “How interested would you be in doing genetic testing to learn about your risk of developing a cancer that may be able to be prevented or treated”) and carrier status information (i.e., “How interested would you be in doing genetic testing to learn about a gene variation that does not affect your health but might affect the health of your children”), as well as testing to learn about the risk of a preventable/treatable disease, risk of an unpreventable/untreatable disease, and medication response. To assess delivery preferences, we also had two items assessing the level of interest in genetic testing as part of a general check-up either “with a health care provider” or “through your gynecologist’s office.” These items were scored on a seven-point Likert scale from “not at all” to “very” interested. The responses were dichotomized as “very” interested vs. all other categories in order to characterize a high level of interest (Kaphingst et al., 2018). A second set of interest items assessed interest in the next year in having the same five types of genetic testing if offered (“If it were offered, would you be interested in having the following types of genetic testing in the next year”). Respondents answered yes, no, or not sure to each item. Responses were dichotomized as yes vs. no/not sure for analysis.

#### 2.2.2 Predictor Variables

Selection of hypothesized predictors was informed by a conceptual framework based on the model of Risk Information and Processing and Uncertainty Management Theory (Griffin et al., 1999; Brashers, 2001).

##### 2.2.2.1 Worry About Genetic Risks

We assessed genetic worry with three items (e.g., “On a scale from 1 to 7 where 1 is not at all worried, and 7 is extremely worried, please describe how worried you are about the following: your genes put you at increased risk for developing a common disease, like heart disease or diabetes”) (Biesecker et al., 2009). Response options were on a seven-point Likert-type scale from “not at all” to “extremely” worried. We calculated an average genetic worry score (Cronbach’s *α* of 0.83), which was treated continuously in analysis.

##### 2.2.2.2 Genetic Self-Efficacy

We assessed genetic self-efficacy (i.e., individuals’ confidence in their ability to use genetic information) using a three-item measure on which participants indicated the extent to which they agreed with each item on a five-point Likert-type scale from strongly disagree to strongly agree (i.e., “I can explain genetic issues to people”) (Parrott et al., 2004). Scores on these items were averaged (Cronbach’s *α* of 0.76) and modeled as a continuous variable in analysis.

##### 2.2.2.3 Genetic Knowledge

To assess general knowledge about genetics, we utilized an 18-item (e.g., “Altered” (mutated) genes can cause disease”) measure (Fitzgerald-Butt et al., 2016). Each item was answered as true, false, or not sure. Correct answers were summed (Cronbach’s α of 0.81) and the sum score was treated as a continuous variable for analysis.

##### 2.2.2.4 Importance of Genetic Information

We used two items to assess the perceived importance of genetic information, one focused on cancer predisposition testing (i.e., “Please mark how important it is to you to learn more about how your genes may affect your chance of getting cancer”) and one on carrier screening, adapted from our prior work (McBride et al., 2009; Kaphingst et al., 2016b). Both items were answered on a seven-point scale from “not at all important” to “very important.” Responses were dichotomized (Cronbach’s *α* of 0.69) as very important vs. other categories for analysis.

##### 2.2.2.5 Health Consciousness

Participants’ degree of health consciousness was assessed with five items (e.g., “my health depends on how well I take care of myself”), which were answered on a five-point Likert-type scale from “strongly disagree” to “strongly agree” (Dutta-Bergman, 2003). The responses were averaged (Cronbach’s *α* of 0.83) and treated continuously in analysis. Higher scores indicated a stronger health consciousness.

##### 2.2.2.6 Health Information Orientation

The importance placed on health information was assessed with eight items (e.g., “It is important to me to be informed about health issues”), which were answered on a five-point Likert-type scale from “strongly disagree” to “strongly agree” (Dutta-Bergman, 2003). The responses were averaged (Cronbach’s *α* of 0.86) and treated continuously in analysis. Higher scores indicated a stronger health information orientation.

##### 2.2.2.7 Health Information Seeking

One item was used to assessed health information seeking (i.e., “In the past 30 days, how often would you say you have looked for information about ways to stay healthy or to feel better?“), which respondents answered on a four-point Likert-type scale from “Not at all” to “Very often” (Kaphingst et al., 2012; National Cancer Institute, 2015). Responses were treated as categorical in analysis.

##### 2.2.2.8 Risk Perceptions

We assessed relative risk perceptions for breast, ovarian, and colon cancer with three items (e.g., “Based on this information, compared to most people your age and sex, would you say that you are…,)” which was answered on a five-point scale from “a lot less likely” to “a lot more likely” to get the disease (Wertz et al., 1986; Lipkus et al., 2000). Risk perceptions were treated dichotomized as “somewhat” or “a lot” more likely vs. other categories for analysis.

##### 2.2.2.9 Social Influences

We assessed social influences on learning more about health (i.e., normative beliefs) and motivation to comply using two items from our prior research (Hay et al., 2012): “The people who mean the most to me think I should learn more about ways I can keep myself healthy” and “On a scale from 1 to 7 where 1 is not at all motivated and 7 is very motivated, how motivated you would say you are to do what these people want you to do?” These items were answered on seven-point Likert-types scales from “strongly agree” to “strongly disagree” and “not at all” to “very” motivated, respectively. Responses (Cronbach’s *α* of 0.72) were dichotomized as strongly agree or very motivated vs. other categories for analysis.

##### 2.2.2.10 Intolerance for Uncertainty

We utilized the 12-item short version of the intolerance of uncertainty scale (i.e., “I always want to know what the future has in store for me”) (Carleton et al., 2007). Respondents answered each item on a five-point Likert-type scale from “Not at all” to “Entirely” characteristic of me. Following scoring rules, we summed the responses (Cronbach’s α of 0.89) and treated as continuous in analysis.

##### 2.2.2.11 Numeracy

We assessed numeracy using the Subjective Numeracy Scale, a self-report measure with two four-item subscales: perceived ability to perform mathematical tasks and preference for the use of numeric versus verbal information (Fagerlin et al., 2007). Each item was answered on a six-point Likert-type scale (e.g., “not at all good” to “extremely good” and “always prefer words” to “always prefer numbers/percentages”). Following standard scoring, we averaged the responses (Cronbach’s *α* of 0.85), and treated the average score as continuous in analysis. Higher scale scores reflected greater perceived ability and stronger preference for numeric information.

##### 2.2.2.12 Health Literacy

Health literacy was assessed with a three-item screener (e.g., “How confident are you filling out medical forms by yourself?”) (Chew et al., 2008). Each item was answered on five-point Likert-type scales. Responses were summed and treated as continuous in analysis.

#### 2.2.3 Sociodemographic Characteristics

We also assessed the following characteristics as potential covariates: age, race, ethnicity, Jewish ancestry, educational attainment, marital status, having biological children, planning to become pregnant in next year, urban vs. rural residence, household income, health insurance status, personal history of cancer, family history of cancer, and having had prior genetic testing.

### 2.3 Analysis

Descriptive statistics were calculated for each variable. We used chi-squared tests to evaluate whether Latina women differed from non-Latina women in their level of interest in various types of genetic testing. Because of sociodemographic differences by ethnicity, we also examined the effect of Latina ethnicity in multivariable logistic regression models. To identify potential predictors of interest in cancer predisposition testing and carrier status testing, which were the areas of focus for this analysis, we used chi-squared tests for associations with categorical variables, t-tests for continuous variables, and the Wilcoxon Rank Sum Test for non-normal continuous variables. Of these predictors, those with a bivariate association of *p* < 0.10 were included in multivariable logistic regression models (Hildalgo and Goodman, 2013). Sociodemographic covariates (i.e., age, race, Jewish ancestry, educational attainment, marital status, having biological children, planning to become pregnant in the next year, urban vs. rural residence, household income, health insurance status, personal history of cancer, family history of cancer, having had prior genetic testing) were also assessed in these models, and those covariates with a *p* < 0.10 were retained in final multivariable logistic regression models. An interaction variable between ethnicity and intolerance for uncertainty was also tested for entry in these models. However, since the interaction term was not significant in any of the models we present the final models without the interaction term. We re-fit the final multivariable models on samples stratified by ethnicity to examine whether predictors of the interest outcome variables were the same for Latina vs. non-Latina women. For final models, we present odds ratios along with their corresponding 95% confidence intervals. R was used for all analyses (R Core Team, 2019). The statistical significance level was set at *p* < 0.05.

## 3 Results

### 3.1 Participant Characteristics

The mean age of respondents was 25.7 years (SD = 4.8). About 50% of respondents identified as white/Caucasian, 39% as Latina/Hispanic, and 24% as Black/African-American. The majority had not completed college; 29% had a high school degree or less and 40% had some college education. Respondents had a moderate level of self-reported numeracy ability (M = 3.9; SD = 1.2) and health literacy (M = 9.5; SD = 1.8). About half (50%) had a household income of <$50,000. Less than half were married or living as married (40%). About 47% had biological children, and 28% reported that they were planning to become pregnant in the next year. Few respondents (10%) reported a personal history of cancer, although 58% had a family history of cancer. Less than half (31%) reported having had prior genetic testing. As shown in Table 1, having had biological children, race, having Ashkenazi Jewish ancestry, planning to become pregnant in the next year, rural vs. urban residence, having had genetic testing, having a personal history of cancer, and age differed significantly between Latina and non-Latina respondents.

**TABLE 1:** Sociodemographic characteristics of 450 female respondents by ethnicity.

Characteristics	*Latina*/Hispanic (*n* = 176)		Non-Hispanic/non-*Latina*/other (*n* = 274)		*p*-value
	*N*	%	*N*	%	
Educational attainment					0.29
High school degree/junior high	44	25.1	86	31.5	
Some college/associate degree	76	43.4	102	37.4	
College degree or higher	55	31.4	85	31.1	
Married/living as married	72	41.1	104	38.4	0.63
Have biological children	94	53.7	114	41.9	**0.019**
Race					**<0.001**
White/Caucasian	77	44.0	147	53.8	
Black/African-American	26	14.9	83	30.4	
Asian/Pacific Islander/Native Hawaiian	16	9.1	23	8.4	
Multi-racial	22	12.6	19	7.0	
Other	34	19.4	1	0.4	
Have Ashkenazi (Eastern European) Jewish ancestry	46	26.3	30	11.1	**<0.001**
Planning to become pregnant in the next year					**0.048**
Yes	60	34.5	65	23.8	
No	85	48.9	158	57.9	
Not sure	29	16.7	50	18.3	
Geographic location					**<0.001**
Urban	11	6.4	56	21.1	
Rural/Frontier	161	93.6	210	78.9	
Household income					0.067
<$25,000	38	21.7	82	29.9	
$25,000–$49,999	44	25.1	62	22.6	
$50,000–$74,999	43	24.6	57	20.8	
>$74,999	46	26.3	56	20.4	
Prefer not to answer	4	2.3	17	6.2	
Health insurance					0.17
Private insurance	89	50.9	126	46.2	
Public insurance	66	37.7	98	35.9	
No	20	11.4	49	17.9	
Have had genetic testing	60	37.7	63	26.2	**0.02**
Have personal history of cancer	27	15.3	19	7.0	**0.007**
Have family history of cancer	90	57.0	141	58.0	0.915
	Mean	SD	Mean	SD	
Current age	25.0	4.5	26.1	5.0	**0.02**

SD, standard deviation.

In terms of possible psychosocial predictors of interest in genetic testing (Table 2), approximately 27% of participants reported that cancer genetic information was very important to them and 35% thought that carrier status information was very important. Most (65%) sought health information either somewhat often or very often. About half of respondents believed that important others strongly valued keeping oneself healthy (48%). Respondents had a moderate level of health consciousness (M = 3.7; SD = 0.9), health information orientation (M = 3.6; SD = 0.8), and intolerance for uncertainty (M = 39.8; SD = 10.1). Less than one-third perceived themselves as more likely to develop breast (24%), ovarian (32%), or colon (31%) cancer than the average woman of their race. They had moderate worry about their genetic risks (M = 4.3; SD = 1.6), and a moderate degree of genetic self-efficacy (M = 9.5; SD = 3.1) and genetic knowledge (M = 9.1; SD = 4.3).

**TABLE 2:** Psychosocial characteristics of 450 female respondents.

Characteristics	*N*	%
High importance of cancer genetic information (*n =* 449)	121	26.9
High importance of carrier status information (*n =* 449)	155	34.5
Health information seeking (n = 447)		
Very often	104	23.3
Somewhat often	186	41.6
Not very often	126	28.2
Not at all	31	6.9
Risk perception (Somewhat more likely/a lot more likely)		
Breast cancer (*n =* 445)	108	24.3
Ovarian cancer (*n =* 446)	142	31.8
Colon cancer (*n =* 446)	139	31.2
Strongly agree that the people who mean the most to me think I should learn more about ways I can keep myself healthy. (*n =* 447)	214	47.9
Very motivated to do what these people want you to do. (*n =* 447)	194	43.4
	Mean (SD)	Range
Numeracy Ability subscale (*n =* 446)	3.9 (1.2)	1–6
Numeracy Preference subscale (*n =* 447)	3.9 (1.1)	1–6
Health Literacy (*n =* 450)	9.5 (1.8)	0–13
Worry about genetic risks (*n =* 450)	4.3 (1.6)	1–7
Genetic self-efficacy (*n =* 450)	9.5 (3.1)	0–15
Genetic knowledge (*n =* 450)	9.1 (4.3)	0–18
Health consciousness (*n =* 448)	3.7 (0.9)	1–5
Health information orientation (*n =* 448)	3.6 (0.8)	1–5
Intolerance for uncertainty (*n =* 450)	39.8 (10.1)	0–60

SD, standard deviation.

### 3.2 Interest in Different Types of Genetic Testing

We assessed how interested respondents would be in having each type of genetic testing in the next year if it were offered (Table 3). More than half reported that they would be interested in receiving genetic testing in the next year to learn information about carrier status (56%), risk of a preventable or treatable disease (55%), and medication response (53%). A slightly lower proportion reported that they would be interested in receiving genetic testing to learn about their risk of a preventable or treatable cancer (49%), and the lowest level of interest was in having genetic testing to learn about the risk of an unpreventable or untreatable disease (45%).

**TABLE 3:** Interest in cancer predisposition testing and carrier screening among respondents (*n* = 450).

Outcome	*N*	%
Very interested in genetic testing as part of a general check-up		
With your health care provider (*n =* 450)	110	24.4
Through your gynecologist’s office (*n =* 450)	103	22.9
Very interested in genetic testing to learn about		
Your risk of developing a disease that may be able to be prevented or treated (*n =* 447)	110	24.6
Your risk of developing a *cancer* that may be able to be prevented or treated (*n =* 447)	116	26.0
Your risk of developing a disease that cannot be prevented or treated (*n =* 447)	87	19.5
How you would respond to a medication for a disease (*n =* 447)	95	21.3
A gene variation that does not affect your health but might affect the health of your children (*n =* 447)	119	26.6
Yes, Interested in having the following types of genetic testing in the next year		
Your risk of developing a disease that may be able to be prevented or treated (*n =* 450)	246	54.7
Your risk of developing a *cancer* that may be able to be prevented or treated (*n =* 449)	222	49.4
Your risk of developing a disease that cannot be prevented or treated (*n =* 450)	203	45.1
How you would respond to a medication for a disease (*n =* 449)	239	53.2
A gene variation that does not affect your health but might affect the health of your children (*n =* 449)	249	55.5

SD, standard deviation.

To further investigate women’s level of interest in genetic testing, we also examined the proportion of respondents having a high level of interest (i.e., reporting being “very interested”). When asked about genetic testing as part of a general check-up, 24% were very interested in receiving testing with their healthcare provider and 23% through a gynecologist. For different types of testing, we found the highest proportions were very interested in genetic testing to learn about their risk of developing a preventable or treatable cancer (26%) and learn about carrier status (27%). Similarly, about 25% were very interested in learning about their risk of preventable or treatable diseases more generally. A slightly lower proportion reported being very interested in genetic testing to learn about pharmacogenomic variants (21%) or risk of an unpreventable or untreatable disease (20%).

### 3.3 Differences in Interest in Testing by Ethnicity

Interest in different types of testing was generally similar between Latina respondents and non-Latina respondents, as was interest in genetic testing as part of a general check-up (Table 4). However, for interest in having genetic testing in the next year if offered, we found that Latina respondents (60.8%) were more likely to say that they would be interested in testing for risk of a preventable or treatable disease than non-Latina respondents (51%; *p* = 0.046). For level of interest in different types of genetic testing, we found that Latina respondents (32.0%) were more likely to be very interested in learning about their risk of a preventable or treatable cancer compared with non-Latina respondents (22.1%; *p* = 0.03). Latina respondents (25.7%) were also more likely to be very interested in learning about their risk of an unpreventable or untreatable disease compared with non-Latina respondents (15.4%; *p* = 0.01). There was a trend toward a greater proportion being very interested in carrier status information (32.0% among Latina participants vs. 23.2% among non-Latina participants, *p* = 0.051).

**TABLE 4:** Bivariate associations between genetic testing interest and ethnicity (*n* = 450).

		Latina	Non-Latina	*p*-value
		*n* = 176	*n* = 274	
Interest in genetic testing to learn about				
Your risk of developing a disease that may be able to be prevented or treated	Very interested	51 (29.1)	59 (21.7)	*0.094*
	Other categories	124 (70.9)	213 (78.3)	
Your risk of developing a *cancer* that may be able to be prevented or treated	Very interested	56 (32.0)	60 (22.1)	**0.026**
	Other categories	119 (68.0)	212 (77.9)	
Your risk of developing a disease that cannot be prevented or treated	Very interested	45 (25.7)	42 (15.4)	**0.011**
	Other categories	130 (74.3)	230 (84.6)	
How you would respond to a medication for a disease	Very interested	41 (23.4)	54 (19.9)	0.43
	Other categories	134 (76.6)	218 (80.1)	
A gene variation that does not affect your health but might affect the health of your children	Very interested	56 (32.0)	63 (23.2)	*0.051*
	Other categories	119 (68.0)	209 (76.8)	
Interest in genetic testing as part of a general check-up				
With your health care provider	Very Interested	47 (26.7)	63 (23.0)	0.43
	Other categories	129 (73.3)	211 (77.0)	
Through your gynecologist’s office	Very Interested	48 (27.3)	55 (20.1)	*0.097*
	Other categories	128 (72.7)	219 (79.9)	
Interested in having the following types of genetic testing in the next year				
Your risk of developing a disease that may be able to be prevented or treated	Yes	107 (60.8)	139 (50.7)	**0.046**
	No/Not sure	69 (39.2)	135 (49.3)	
Your risk of developing a *cancer* that may be able to be prevented or treated	Yes	89 (50.6)	133 (48.7)	0.78
	No/Not sure	87 (49.4)	140 (51.3)	
Your risk of developing a disease that cannot be prevented or treated	Yes	87 (49.4)	116 (42.3)	0.17
	No/Not sure	89 (50.6)	158 (57.7)	
How you would respond to a medication for a disease	Yes	99 (56.6)	140 (51.1)	0.30
	No/Not sure	76 (43.4)	134 (48.9)	
A gene variation that does not affect your health but might affect the health of your children	Yes	103 (58.9)	146 (53.3)	0.29
	No/Not sure	72 (41.1)	128 (46.7)	

*p*-value by Chi-square Test; Significant results are bolded.

### 3.4 Bivariate Predictors of Interest in Genetic Testing

We next examined the bivariate relationships of hypothesized predictors and ethnicity with interest in genetic testing for cancer predisposition and carrier status. As shown in Table 5, being interested in both types of genetic testing in the next year if it were offered was associated with higher worry about genetic risks (both *p* < 0.001), higher genetic self-efficacy (both *p* < 0.05), higher genetic knowledge (both *p* < 0.001), greater perceived importance of cancer genetic information (both *p* < 0.001) and carrier status information (both *p* < 0.001), greater health consciousness (both *p* < 0.001), stronger health orientation (both *p* < 0.001), greater health information seeking (both *p* < 0.05), stronger social influence (both *p* < 0.001), higher intolerance for uncertainty (both *p* < 0.001), and higher subjective numeracy (both *p* < 0.001). Higher breast cancer risk perceptions were significantly associated with interest in cancer predisposition testing (*p* < 0.05) but not carrier status testing, and ovarian and colorectal cancer risk perceptions were not significantly related with interest in either type of genetic testing in the next year.

**TABLE 5:** Bivariate predictors of interest in receiving cancer predisposition testing and carrier screening (*n* = 450).

Predictor	Cancer predisposition testing		Carrier status	
	Very interested^a^	Yes, interested in next year^b^	Very interested^a^	Yes, interested in next year^b^
	*n = 116*	*n = 222*	*n = 119*	*n = 249*
Worry about genetic risks, median [IQR]	**6.0 [4.0–7.0]**	**5.0 [3.8–6.3]**	**5.7 [4.0–7.0]**	**5.0 [3.7–6.3]**
Genetic self-efficacy, mean (SD)	**10.4 (3.9)**	**9.8 (3.3)**	**10.3 (3.8)**	**9.8 (3.2)**
Genetic knowledge, median [IQR]	**11.0 [8.0–13.0]**	**11.0 [9.0–14.0]**	**11.0 [8.0–13.0]**	**11.0 [7.0–13.0]**
Importance of cancer genetic information, n (%)				
Very important	**76 (65.5)**	**88 (39.6)**	**72 (60.5)**	**91 (36.5)**
Other categories	**40 (34.5)**	**134 (60.4)**	**47 (39.5)**	**158 (63.5)**
Importance of carrier status information, n (%)				
Very important	**91 (78.4)**	**110 (49.5)**	**86 (72.3)**	**115 (46.2)**
Other categories	**25 (21.6)**	**112 (50.5)**	**33 (27.7)**	**134 (53.8)**
Health consciousness, median [IQR]	**4.4 [4.0–5.0]**	**4.0 [3.4–4.6]**	**4.2 [3.6–5.0]**	**3.8 [3.2–4.4]**
Health orientation, median [IQR]	**4.4 [3.8–4.9]**	**3.9 [3.3–4.5]**	**4.3 [3.6–4.9]**	**3.8 [3.1–4.4]**
Health information seeking, n (%)				
Very often	**47 (40.5)**	**62 (27.9)**	**48 (40.3)**	**68 (27.4)**
Somewhat often	**46 (39.7)**	**93 (41.9)**	**47 (39.5)**	**104 (41.9)**
Not very often	**17 (14.7)**	**57 (25.7)**	**16 (13.4)**	**59 (23.8)**
Not at all	**6 (5.2)**	**10 (4.5)**	**8 (6.7)**	**17 (6.9)**
Risk perceptions				
Breast cancer, n (%)				
Somewhat more likely/a lot more likely	**45 (38.8)**	**65 (29.5)**	**48 (40.3)**	69 (27.8)
About as likely	**38 (32.8)**	**80 (36.4)**	**34 (28.6)**	88 (35.5)
A lot less likely/somewhat less likely	**33 (28.4)**	**75 (34.1)**	**37 (31.1)**	91 (36.7)
Ovarian cancer, n (%)				
Somewhat more likely/a lot more likely	**50 (43.1)**	73 (33.0)	**51 (42.9)**	80 (32.3)
About as likely	**37 (31.9)**	81 (36.7)	**34 (28.6)**	93 (37.5)
A lot less likely/somewhat less likely	**29 (25.0)**	67 (30.3)	**34 (28.6)**	75 (30.2)
Colon cancer, n (%)				
Somewhat more likely/a lot more likely	35 (30.2)	61 (27.6)	32 (26.9)	72 (29.0)
About as likely	31 (26.7)	74 (33.5)	35 (29.4)	75 (30.2)
A lot less likely/somewhat less likely	50 (43.1)	86 (38.9)	52 (43.7)	101 (40.7)
Motivation				
Normative beliefs, n (%)				
Strongly Agree	**91 (78.4)**	**141 (63.5)**	**89 (74.8)**	**141 (56.9)**
Other categories	**25 (21.6)**	**81 (36.5)**	**30 (25.2)**	**107 (43.1)**
Motivation to comply, n (%)				
Very motivated	**79 (68.1)**	**118 (53.2)**	**79 (66.4)**	**125 (50.4)**
Other categories	**37 (31.9)**	**104 (46.8)**	**40 (33.6)**	**123 (49.6)**
Intolerance for uncertainty, mean (SD)	**45.1 (10.9)**	**41.6 (10.0)**	**44.4 (11.0)**	**41.5 (10.3)**
Subjective numeracy, mean (SD)	**4.5 (1.1)**	**4.2 (1.0)**	**4.4 (1.2)**	**4.1 (1.1)**

Bold indicates *p* < 0.05; SD: standard deviation; IQR: interquartile range; *p*-value by Wilcoxon Rank Sum Test for the following variables: Worry about genetic risks, Genetic knowledge, Health consciousness, and Health orientation; *p*-value by T-test for Genetic self-efficacy, Intolerance for uncertainty, and Subjective numeracy; *p*-value by Chi-squared Test for: Importance of cancer genetic information, Importance of carrier status information, Health information seeking, and Risk perceptions (breast, ovarian, and colon cancers).

a Very interested vs. other categories.

b Yes vs. no/not sure.

We found similar patterns of bivariate associations for the outcome of being very interested in genetic testing, with the exception of risk perceptions. Being very interested in both types of genetic testing was associated with higher worry about genetic risks (both *p* < 0.001), higher genetic self-efficacy (both *p* < 0.001), higher genetic knowledge (both *p* < 0.001), greater perceived importance of cancer genetic information (both *p* < 0.001) and carrier status information (both *p* < 0.001), greater health consciousness (both *p* < 0.001), stronger health orientation (both *p* < 0.001), greater health information seeking (both *p* < 0.001), higher breast cancer risk perceptions (both *p* < 0.001), higher ovarian cancer risk perceptions (both *p* < 0.01), stronger social influence (both *p* < 0.001), higher intolerance for uncertainty (both *p* < 0.001), and higher subjective numeracy (both *p* < 0.001).

### 3.5 Multivariable Predictors of Interest in Genetic Testing

In multivariable logistic regression models, Latina ethnicity was not associated with any interest outcome (Table 6). In multivariable models, respondents who were interested in being tested for cancer predisposition in the next year had higher worry about genetic risks (OR = 1.44; 95% CI: 1.22–1.72) and higher genetic knowledge (OR = 1.26; 95% CI: 1.18–1.35). They were also more likely to report that they did not seek health information very often compared to those who said not at all (OR = 2.97; 95% CI: 1.05–8.93). Respondents who were interested in receiving carrier screening in the next year had also higher worry about genetic risks (OR = 1.39; 95% CI: 1.17–1.64) and higher genetic knowledge (OR = 1.11; 95% CI: 1.05–1.18). They were also more likely to perceive carrier status information as very important (OR = 2.46; 95% CI: 1.24–4.97), although those with lower health consciousness were more interested in genetic testing for carrier status (OR = 0.60; 95% CI: 0.39–0.91).

**TABLE 6:** Multivariable logistic regression models showing predictors of interest in receiving cancer predisposition testing and carrier screening.

Tested predictors	Cancer predisposition testing		Carrier status	
	Very interested^a^ (*n* = 440)	Yes, interested in next year^b^ (*n* = 442)	Very interested^a^ (*n* = 442)	Yes, interested in next year^b^ (*n* = 431)
	OR (95% CI)	OR (95% CI)	OR (95% CI)	OR (95% CI)
Worry about genetic risks	**1.29 (1.06, 1.57)**	**1.44 (1.22, 1.72)**	1.12 (0.93, 1.35)	**1.39 (1.17, 1.64)**
Genetic self-efficacy	0.95 (0.86, 1.05)	1.01 (0.92, 1.10)	0.98 (0.89, 1.08)	0.98 (0.90, 1.07)
Genetic knowledge	1.02 (0.94, 1.11)	**1.26 (1.18, 1.35)**	1.09 (1.00, 1.18)	**1.11 (1.05, 1.18)**
Importance of cancer genetic information	**2.71 (1.35, 5.46)**	1.44 (0.72, 2.90)	**2.58 (1.29, 5.15)**	1.21 (0.61, 2.42)
Importance of carrier status information	**3.53 (1.69, 7.44)**	1.53 (0.78, 3.02)	**3.00 (1.44, 6.30)**	**2.46 (1.24, 4.97)**
Health consciousness	**1.89 (1.06, 3.42)**	0.82 (0.54, 1.24)	0.95 (0.55, 1.63)	**0.60 (0.39, 0.91)**
Health orientation	1.21 (0.65, 2.29)	1.47 (0.90, 2.42)	1.31 (0.73, 2.41)	1.46 (0.91, 2.37)
Health information seeking^c^				
Not very often	0.64 (0.17, 2.58)	**2.97 (1.05, 8.93)**	0.28 (0.08, 1.01)	0.68 (0.26, 1.75)
Somewhat often	0.60 (0.17, 2.32)	2.03 (0.73, 5.98)	0.40 (0.13, 1.33)	0.70 (0.27, 1.80)
Very often	0.60 (0.15, 2.49)	1.78 (0.58, 5.67)	0.53 (0.15, 1.95)	0.72 (0.24, 2.08)
Breast Cancer Risk Perception^d^				
About as likely	1.32 (0.54, 3.29)	1.21 (0.71, 2.07)	0.98 (0.45, 2.17)	
Somewhat more likely/a lot more likely	1.53 (0.60, 3.92)	1.29 (0.70, 2.38)	1.95 (0.85, 4.51)	
Ovarian Cancer Risk Perception^d^				
About as likely	1.15 (0.46, 2.84)		0.83 (0.37, 1.86)	
Somewhat more likely/a lot more likely	1.29 (0.48, 3.46)		1.17 (0.49, 2.80)	
Normative beliefs	1.34 (0.64, 2.78)	1.69 (0.99, 2.91)	1.48 (0.76, 2.88)	1.08 (0.63, 1.83)
Motivation to comply	1.03 (0.50, 2.11)	0.80 (0.45, 1.40)	1.24 (0.64, 2.38)	1.08 (0.62, 1.86)
Intolerance for uncertainty	1.00 (0.96, 1.04)	0.98 (0.95, 1.01)	1.00 (0.97, 1.04)	1.00 (0.97, 1.03)
Subjective numeracy	1.32 (0.94, 1.86)	1.18 (0.89, 1.58)	0.99 (0.71, 1.36)	1.13 (0.85, 1.50)
Covariates				
Non-Hispanic/Non-Latina/Other^e^	0.56 (0.30, 1.04)	0.97 (0.60, 1.58)	0.64 (0.36, 1.14)	1.01 (0.63, 1.62)
Health Literacy	**1.38 (1.13, 1.70)**			
Educational attainment^f^				
Some college/associate degree			**0.46 (0.22, 0.96)**	1.52 (0.87, 2.68)
College degree or higher			1.20 (0.55, 2.63)	1.07 (0.56, 2.02)
Household income^g^				
$25,000–$49,999			0.69 (0.31, 1.52)	**2.42 (1.30, 4.57)**
$50,000–$74,999			2.02 (0.90, 4.58)	**2.31 (1.19, 4.58)**
>$74,999			1.09 (0.47, 2.54)	1.90 (0.96, 3.77)
Prefer not to answer			0.15 (0.01, 0.94)	2.74 (0.91, 8.66)
Geographic location: Urban^h^				**2.02 (1.06, 3.88)**
Health Insurance^i^				
Public insurance				1.15 (0.58, 2.29)
Private insurance				1.59 (0.80, 3.19)

Significant results are bolded.

a Very interested vs. other categories.

b Yes vs. no/not sure.

c Compared with not at all.

d Compared with a lot less likely/somewhat less likely.

e Compared with Latina/Hispanic.

f Compared with High school degree/junior high.

g Compared with <$25,000.

h Compared with Rural/Frontier.

i Compared with no insurance.

For the outcome of high level of interest, being very interested in genetic testing for cancer predisposition was associated with higher worry about genetic risks (OR = 1.29; 95% CI: 1.06–1.57), higher perceived importance of cancer genetic information (OR = 2.71; 95% CI: 1.35–5.46), higher perceived importance of carrier status information (OR = 3.53; 95% CI: 1.69–7.44), and higher health literacy (OR = 1.38; 95% CI: 1.13–1.70). Being very interested in genetic testing for carrier status was associated with higher perceived importance of cancer genetic information (OR = 2.57; 95% CI: 1.29–5.12) and higher perceived importance of carrier status information (OR = 3.00; 95% CI: 1.44–6.29). In this model, respondents with some college were less likely to report being very interested than those with a high school degree (OR = 0.46; 95% CI: 0.22–0.95).

In models stratified by Latina ethnicity, predictors of interest in having cancer predisposition genetic testing in the next year were similar between strata (Table 7), although normative beliefs were a predictor of interest only among non-Latina respondents (OR = 2.02; 95% CI: 1.01–4.07). For predictors of interest in testing to learn carrier status information, worry about genetic risks was a significant predictor in both strata. However, higher genetic knowledge was a predictor of interest among Latina women (OR = 3.06; 95% CI: 1.33–7.38), and greater importance of carrier status information and income were predictors only among non-Latina respondents (OR = 2.82; 95% CI: 1.27–6.45). For predictors of a high level of interest in genetic testing (Table 8), higher worry about genetic risks was a significant predictor of being very interested in cancer predisposition testing only among non-Latina respondents (OR = 1.38; 95% CI: 1.06–1.79). Higher perceived importance of cancer genetic information was a significant predictor of being very interested in both cancer predisposition testing (OR = 3.85; 95% CI: 1.24–11.88) and carrier screening (OR = 3.60; 95% CI: 1.10–11.82) among Latina respondents, while higher perceived importance of carrier status information was related to these outcomes among non-Latina respondents (OR = 7.53; 95% CI: 2.64–21.46 and OR = 3.34; 95% CI: 1.32–8.43, respectively).

**TABLE 7:** Multivariable logistic regression models, stratified by ethnicity, showing predictors of interest in receiving cancer predisposition testing and carrier screening in the next year.

Tested predictors	Interest in receiving genetic testing for cancer predisposition testing in next year^a^		Interest in receiving genetic testing for carrier status in next year^a^	
	*Latina*/Hispanic (*n* = 170)	Non-Hispanic/non-*latina*/other (*n* = 272)	Latina/hispanic (*n* = 168)	Non-hispanic/non-latina/other (*n* = 263)
	OR (95% CI)	OR (95% CI)	OR (95% CI)	OR (95% CI)
Worry about genetic risks	**1.68 (1.22, 2.40)**	**1.34 (1.09, 1.66)**	**1.48 (1.08, 2.08)**	**1.35 (1.10, 1.67)**
Genetic self-efficacy	1.03 (0.87, 1.23)	1.00 (0.89, 1.11)	1.05 (0.89, 1.23)	0.92 (0.83, 1.03)
Genetic knowledge	**1.28 (1.13, 1.47)**	**1.25 (1.15, 1.36)**	**1.14 (1.02, 1.28)**	1.07 (1.00, 1.15)
Importance of cancer genetic information	1.46 (0.41, 5.02)	1.47 (0.61, 3.55)	0.69 (0.20, 2.27)	1.35 (0.56, 3.30)
Importance of carrier status information	1.31 (0.38, 4.59)	1.41 (0.62, 3.27)	1.61 (0.45, 6.02)	**3.06 (1.33, 7.38)**
Health consciousness	1.00 (0.45, 2.19)	0.82 (0.49, 1.37)	0.80 (0.37, 1.67)	0.62 (0.36, 1.04)
Health orientation	1.48 (0.61, 3.74)	1.50 (0.81, 2.81)	1.90 (0.81, 4.63)	1.32 (0.71, 2.48)
Health information seeking^b^				
Not very often	3.93 (0.44, 88.71)	2.85 (0.86, 10.04)	0.08 (0.00, 0.82)	1.38 (0.46, 4.29)
Somewhat often	2.52 (0.28, 56.86)	1.96 (0.61, 6.68)	0.10 (0.00, 0.99)	1.29 (0.43, 3.97)
Very often	2.38 (0.22, 58.06)	1.79 (0.49, 6.80)	0.09 (0.00, 1.02)	1.61 (0.44, 5.94)
Breast Cancer Risk Perception^c^				
About as likely	1.13 (0.44, 2.92)	1.26 (0.65, 2.44)		
Somewhat more likely/a lot more likely	1.32 (0.46, 3.83)	1.28 (0.58, 2.81)		
Normative beliefs	1.34 (0.52, 3.43)	**2.02 (1.01, 4.07)**	1.23 (0.52, 2.89)	1.03 (0.52, 2.03)
Motivation to comply	1.46 (0.58, 3.59)	0.49 (0.22, 1.04)	0.84 (0.33, 2.04)	1.08 (0.52, 2.21)
Intolerance for uncertainty	0.95 (0.90, 1.01)	0.99 (0.95, 1.03)	1.01 (0.96, 1.07)	0.99 (0.95, 1.03)
Subjective numeracy	0.91 (0.48, 1.69)	1.30 (0.93, 1.83)	0.99 (0.56, 1.69)	1.15 (0.82, 1.62)
Educational attainment^d^				
Some college/associate degree			1.03 (0.38, 2.78)	1.74 (0.87, 3.56)
College degree or higher			0.74 (0.22, 2.42)	1.45 (0.66, 3.20)
Household income^e^				
$25,000-$49,999			1.56 (0.52, 4.76)	**2.82 (1.27, 6.45)**
$50,000-$74,999			2.56 (0.80, 8.48)	1.75 (0.74, 4.19)
>$74,999			1.31 (0.41, 4.25)	2.18 (0.90, 5.36)
Prefer not to answer			3.24 (0.35, 52.01)	1.82 (0.50, 6.79)
Geographic location: Urban^f^			2.44 (0.57, 11.24)	2.04 (0.99, 4.26)
Health Insurance^g^				
Public insurance			1.69 (0.45, 6.75)	1.03 (0.45, 2.39)
Private insurance			2.23 (0.60, 8.80)	1.42 (0.62, 3.29)

Significant results are bolded.

a Yes vs. no/not sure.

b Compared with not at all.

c Compared with a lot less likely/somewhat less likely.

d Compared with High school degree/junior high.

e Compared with <$25,000.

f Compared with Rural/Frontier.

g Compared with no insurance.

**TABLE 8:** Multivariable logistic regression models, stratified by ethnicity, showing predictors of being very interested in cancer predisposition testing and carrier screening.

Tested predictors	Very interested in cancer predisposition testing^a^		Very interested in genetic testing for carrier status^a^	
	*Latina*/Hispanic (*n* = 169)	Non-Hispanic/non-*latina*/other (*n* = 271)	Latina/hispanic (*n* = 169)	Non-hispanic/non-latina/other (*n* = 270)
	OR (95% CI)	OR (95% CI)	OR (95% CI)	OR (95% CI)
Worry about genetic risks	1.18 (0.85, 1.66)	**1.38 (1.06, 1.79)**	1.03 (0.72, 1.47)	1.11 (0.89, 1.38)
Genetic self-efficacy	0.88 (0.75, 1.03)	1.01 (0.88, 1.15)	0.98 (0.82, 1.16)	0.98 (0.87, 1.10)
Genetic knowledge	1.00 (0.87, 1.13)	1.09 (0.98, 1.23)	1.10 (0.97, 1.25)	1.07 (0.97, 1.18)
Importance of cancer genetic information	**3.85 (1.25, 11.88)**	2.37 (0.92, 6.13)	**3.60 (1.10, 11.82)**	1.82 (0.75, 4.39)
Importance of carrier status information	1.41 (0.43, 4.64)	**7.53 (2.64, 21.46)**	2.03 (0.55, 7.41)	**3.34 (1.32, 8.43)**
Health consciousness	1.84 (0.78, 4.35)	1.52 (0.63, 3.67)	0.77 (0.32, 1.87)	1.17 (0.59, 2.35)
Health orientation	1.02 (0.41, 2.51)	1.47 (0.57, 3.79)	1.20 (0.46, 3.11)	1.12 (0.52, 2.39)
Health information seeking^b^				
Not very often	0.91 (0.04, 23.77)	0.70 (0.14, 3.59)	0.58 (0.02, 15.59)	0.29 (0.08, 1.15)
Somewhat often	1.57 (0.06, 38.90)	0.33 (0.07, 1.63)	1.46 (0.06, 37.76)	0.35 (0.10, 1.23)
Very often	1.08 (0.04, 29.43)	0.38 (0.06, 2.23)	2.06 (0.07, 57.66)	0.43 (0.10, 1.81)
Breast cancer risk perception^c^				
About as likely	1.72 (0.47, 6.23)	0.87 (0.25, 3.00)	1.62 (0.45, 5.81)	0.82 (0.32, 2.11)
Somewhat more likely/a lot more likely	2.90 (0.82, 10.26)	0.71 (0.17, 2.93)	**3.99 (1.11, 14.40)**	0.86 (0.29, 2.60)
Ovarian cancer risk perception^c^				
About as likely	1.16 (0.32, 4.19)	0.98 (0.27, 3.58)	0.32 (0.09, 1.15)	1.63 (0.57, 4.64)
Somewhat more likely/a lot more likely	0.78 (0.21, 2.84)	2.45 (0.57, 10.57)	0.51 (0.15, 1.75)	2.61 (0.82, 8.30)
Normative beliefs	1.42 (0.52, 3.85)	0.92 (0.31, 2.75)	0.83 (0.29, 2.39)	1.96 (0.85, 4.50)
Motivation to comply	0.79 (0.29, 2.18)	1.80 (0.61, 5.28)	1.03 (0.37, 2.89)	1.37 (0.57, 3.27)
Intolerance for uncertainty	1.01 (0.95, 1.07)	1.00 (0.95, 1.05)	1.04 (0.97, 1.10)	0.99 (0.95, 1.03)
Subjective numeracy	1.12 (0.64, 1.95)	1.23 (0.79, 1.94)	0.83 (0.46, 1.47)	1.01 (0.69, 1.48)
Health Literacy	**1.36 (1.03, 1.79)**	1.29 (0.97, 1.73)		
Educational attainment^d^				
Some college/associate degree			0.41 (0.13, 1.26)	0.73 (0.29, 1.82)
College degree or higher			1.38 (0.38, 5.00)	1.11 (0.42, 2.98)
Household income^e^				
$25,000-$49,999			0.72 (0.20, 2.61)	0.74 (0.27, 2.02)
$50,000-$74,999			1.50 (0.42, 5.40)	1.88 (0.65, 5.46)
>$74,999			0.97 (0.27, 3.54)	1.22 (0.41, 3.65)
Prefer not to answer			0.14 (0.00, 8.28)	0.43 (0.06, 2.90)

Significant results are bolded.

a Very interested vs. other categories.

b Compared with not at all.

c Compared with a lot less likely/somewhat less likely.

d Compared with High school degree/junior high.

e Compared with <$25,000.

## 4 Discussion

In this study, we examined interest, and predictors of interest, in carrier screening and cancer predisposition testing offered as part of routine care among an ethnically diverse sample of 450 women aged 20–35. We found substantial interest in both types of genetic testing, with about half of respondents reporting that they would have each type of testing in the next year if it were offered. The proportion interested in testing for carrier status is consistent with the proportions found to be interested in a hypothetical expanded carrier screening test in prior studies (van Steijvoort et al., 2020; Nijmeijer et al., 2019). The findings also add to our knowledge about interest in cancer predisposition testing in this population if conducted as part of routine clinical care, indicating support from survey respondents for offering genetic testing as part of routine clinical care. Little prior research has examined interest in population-based genetic testing as part of routine care, although in one prior survey conducted in the Netherlands about half of respondents preferred that pre-pregnancy cancer screening be offered via a general practitioner (Plantinga et al., 2016) and another survey found that participants felt that offering personal genomic risk information to the general population to inform prevention and early detection recommendations is acceptable (Smit et al., 2020).

Of note, however, about half of respondents were not interested in testing in the next year, or were not sure, and many did not indicate the highest level of interest in either type of genetic test. It is therefore critical to develop effective decision support tools so that women can make informed decisions about testing if population-based genetic testing efforts are initiated. Better understanding of the predictors of interest is essential to developing effective decision support tools. Consistent with our prior research conducted with women who had been diagnosed with breast cancer at a young age, we found that women’s worry about their broader genetic risks was an important predictor of interest in genetic testing. Notably, worry about genetic risks was predictive, while risk perceptions for breast, ovarian, and colorectal cancer were not predictive of interest in either type of genetic testing in multivariable models. This finding suggests the importance of focusing on information that could be provided about inherited risks, rather than disease risks more generally, in approaches to informed decision making. Also consistent with our prior work, as well as other studies (Kaphingst et al., 2018; Ong et al., 2018), those with higher genetic knowledge were more likely to be interested in both types of testing in the next year. These findings indicate determining key components of genetic knowledge and providing information about these topics is also important in decisional support so that individuals can make informed decisions about genetic testing.

Unlike the findings from our prior work with women who had been diagnosed with breast cancer at a young age (Kaphingst et al., 2018), in this population health information orientation was not predictive of any interest outcomes. Instead, perceived importance of genetic information, either for cancer predisposition testing or carrier status, was related to a number of the interest outcomes. This finding suggests that this general population, which was unselected for personal or family history of disease, may distinguish to a greater extent between genetic information and other types of health information. This hypothesis is also supported by the lack of relationship between health information seeking and interest in genetic testing, suggesting that genetic testing may not be seen as a way to learn more about one’s health and manage health risks, as has been suggested by prior studies conducted in cancer genetic counseling (Rauscher, 2017; Campbell-Salome et al., 2021). In supporting informed decisions about genetic testing as part of routine care, therefore, educational approaches should clearly state what the testing would—and would not—provide in terms of genetic and health risk information.

Neither social influences nor intolerance for uncertainty was predictive of interest in genetic testing for cancer predisposition or carrier status in this population. Our prior research conducted with primary care patients offered genetic susceptibility testing for multiple health conditions had found that social influence from family and friends impacted interest in seeking information about genes (Hay et al., 2012). To explore the importance of social influences further, future research may want to examine different social influences separately. For example, it is possible that interest in genetic testing for carrier status may be more influenced by the normative beliefs of a reproductive partner while interest in testing for cancer predisposition may be more influenced by biological relatives’ beliefs or healthcare providers’ recommendations. Future research may also want to examine whether a measure of how individuals cope with uncertainty about genetic risks specifically is predictive of interest in genetic testing (Biesecker et al., 2017), given the importance of worry about genetic risks observed among our respondents.

Our findings also add to what is known about interest in genetic testing among young Latina women. We generally found similar interest between Latina and non-Latina women in receiving different types of genetic testing in the next year, although a higher proportion of Latina women reported being interested receiving cancer predisposition testing in the next year and being very interested in this type of testing. However, ethnicity was not a significant predictor of interest in multivariable models, suggesting that younger Latina women are just as interested in testing as non-Latina women. We also found many similarities in predictors of interest, such as the importance of worry about genetic risks and genetic knowledge in both strata. These findings suggest the importance of addressing provider- and system-level barriers that may be driving lack of access to and uptake of genetic testing among interested Latina women (Kaphingst et al., 2015; Hay et al., 2019; Turbitt et al., 2019). We also found that perceived importance of different types of genetic information varied by ethnicity. These findings highlight that culturally appropriate approaches to offering genetic services and supporting informed decisions are strongly needed (Gutierrez et al., 2017; French et al., 2018; Shaibi et al., 2018; Srinivasan et al., 2021), particularly if genetic testing were offered to a broad population.

These findings from this study should be considered in light of its limitations. Because population-based genetic testing is not being offered to this population, we asked about interest in hypothetical testing and actual testing uptake is likely to be lower (Persky et al., 2007; Kaphingst et al., 2019). However, predictors of interest are important to developing educational and decision support efforts. We did not specify the cost of testing in the survey items, which could affect responses. The item wording was based on “genetic testing,” but using other terms such as “sequencing” or “screening” could affect level of interest. In addition, we examined interest among potential patients but not providers’ attitudes toward offering genetic testing as part of routine healthcare, and this is an important area for future research. Prior research has indicated that provider support for population-based genetic testing may be more limited (Hann et al., 2017). The sample was a convenience sample and a nationally representative sample would be useful in extrapolating interest to the US population. In addition, the survey was only offered in English, and it will be critical for future studies to examine differences among Spanish-speaking Latina women. Examining the importance of variables such as subethnicity and acculturation will also be important for a fuller understanding of the influence of ethnicity on interest and acceptance of genetic testing (Chavez-Yenter et al., 2021a; Chavez-Yenter et al., 2021b).

## 5 Conclusion

Our findings show substantial interest in both cancer predisposition testing and carrier screening among young women if offered as part of routine healthcare. We found similar interest between Latina and non-Latina women in receiving genetic testing, and worry about genetic risks and genetic knowledge were predictors of interest in both of these groups. The findings showed that women who were more concerned about their genetic risks, had higher knowledge about genetics, and perceived genetic information to be more important were more likely to be interested in both types of genetic testing. These findings therefore indicate support from the survey respondents for offering genetic testing for multiple, clinically indicated genetic tests as part of routine health visits. Such efforts will be important in improving access to genetic information among a broader population of patients than has been reached by many genetic testing initiatives to date. However, it will be critical to develop strategies to standardize outreach to all patients, to develop tools to help healthcare providers offer and communicate about genetic testing, and to address the needs of those who have less prior knowledge about genetics and lower health literacy in order to support informed decision making about genetic testing.

## Data Availability

The data generated for this study is available upon request from the Corresponding Author.
