# Supplementary material for: Interest in Cancer Predisposition Testing and Carrier Screening Offered as Part of Routine Healthcare Among an Ethnically Diverse Sample of Young Women
Source: Front Genet. 2022 Apr 14;13:866062. doi: 10.3389/fgene.2022.866062 (PMC9047995; doi:10.3389/fgene.2022.866062)
Supplement: Supplementary file 1 [file DataSheet1.pdf]

Supplemental File 1. Survey items

Screening questions.

What is your age? ________________

What is your gender?

What is your ethnicity?

- O Latino/Hispanic
- O Non-Hispanic/non-Latino
  - O Other __________________________

Our team is doing a study to better understand women's interest in learning different types of genetic information. We would like to hear your thoughts.

Section 1:

Nowadays, it is possible to predict whether or not a person is likely to develop certain diseases by analyzing their genes. This is called genetic testing. Genetic testing is currently available for a limited number of diseases, but it may be available for more diseases in the future.

Genetic testing could tell you about your risks of different types of diseases.

- **You could learn about your risk of developing a disease that may be able to be prevented or treated.** For example, you could learn that you are more likely to develop colon cancer. In this case, your doctor might recommend that you screen for colon cancer more often.

- **You could learn about your risk of developing a disease that cannot be prevented or treated.** For example, you could learn that you are more likely to develop a muscle disorder that gets worse over time or that you are more likely to develop early dementia from a disease that we don't know how to prevent or treat.

- **You could also learn more about how you respond to a medication for a disease.** For example, you could learn that a certain treatment would not be helpful for you or that you need a different dose of a medicine in order for it to work. You might also learn that you are more or less likely to develop a side effect from a certain treatment.

Another type of information that you might learn from genetic testing **is about a variation in one of your genes that does not affect your health, but could cause health problems if passed on to a child.** For example, you could learn that you have a variation in the gene that causes cystic fibrosis or a variation in the gene that causes sickle cell disease. Having a variation in this type of gene would not cause any health problems for you. But if you were to have children with someone who also had a variation in the same gene, then your children could be born with these serious diseases.

On a scale from 1 to 7, where 1 means not at all interested, and 7 means very interested, if it were offered by a doctor how interested would you be in doing genetic testing to learn about:

	1	2	3	4	5	6	7
	Not at all interested			Somewhat interested			Very interested
Q5_1 Your risk of developing a disease that may be able to be prevented or treated	○ 1	○	○	○ 4	○	○	○ 7
Q5_2 Your risk of developing a <u>cancer</u> that may be able to be prevented or treated	○ 1	○	○	○ 4	○	○	○ 7

Q5_3 Your risk of developing a disease that cannot be prevented or treated	○ 1	○	○	○ 4	○	○	○ 7
Q5_4 How you would respond to a medication for a disease	○ 1	○	○	○ 4	○	○	○ 7
Q5_5 A gene variation that does not affect your health but might affect the health of your children	○ 1	○	○	○ 4	○	○	○ 7

***Q6_1.* On a scale from 1 to 7, where 1 means not at all interested, and 7 means very interested, how interested would you be having any of above types of genetic testing as part of a general check-up with your health care provider?**

Not at all interested			Somewhat interested			Very interested
○	○	○	○	○	○	○
1			4			7

Q6_2 … through your gynecologist's office?

Not at all interested			Somewhat interested			Very interested
○	○	○	○	○	○	○
1			4			7

8. If it were offered by a doctor, would you be interested in having the following types of genetic testing in the next year?

	Yes	No	Not sure
Q8_1 Your risk of developing a disease that may be able to be prevented or treated	○	○	○
Q8_2 Your risk of developing a <u>cancer</u> that may be able to be prevented or treated	○	○	○

Q8_3 Your risk of developing a disease that cannot be prevented or treated	○	○	○
Q8_4 How you would respond to a medication for a disease	○	○	○
Q8_5 A gene variation that does not affect your health but might affect the health of your children	○	○	○

<u>Section 2:</u>

For the next set of questions, we'd like to find out more about how you think about inherited risk.

On a scale from 1 to 7 where 1 is not at all worried, and 7 is extremely worried, please describe how worried you are about the following:

Q9_1 That your genes put you at increased risk for developing a common disease, like heart disease or diabetes.	Not at all worried<br>○<br>1	○	○	Somewhat worried<br>○<br>4	○	○	Extremely worried<br>○<br>7
Q9_2 That you have a genetic variation that means you will certainly develop a disease.	○<br>1	○	○	○<br>4	○	○	○<br>7
Q9_3 That your children could be affected with a genetic condition that you have passed on.	○<br>1	○	○	○<br>4	○	○	○<br>7

On a scale from 1 to 5 where 1 is strongly disagree, and 5 is strongly agree, how much do you agree or disagree with the following statements:

Q10_1 I know how to assess the role of genes for health.	Strongly disagree<br>○<br>1	Somewhat disagree<br>○<br>2	Neither agree nor disagree<br>○<br>3	Somewhat agree<br>○<br>4	Strongly agree<br>○<br>5
Q10_2. I know how to assess my genetic risk for disease.	Strongly disagree<br>○<br>1	Somewhat disagree<br>○<br>2	Neither agree nor disagree<br>○<br>3	Somewhat agree<br>○<br>4	Strongly agree<br>○<br>5

Q10_3 I can explain genetic issues to people.	Strongly disagree<br>○<br>1	Somewhat disagree<br>○<br>2	Neither agree nor disagree<br>○<br>3	Somewhat agree<br>○<br>4	Strongly agree<br>○<br>5

Would you say that the following statements are true or false?

	True	False	Not sure
Q11_1 Some diseases are caused by genes, environment, and lifestyle.	○	○	○
Q11_2 A gene is a disease.	○	○	○
Q11_3 You can see a gene with the naked eye.	○	○	○
Q11_4 Healthy parents can have a child with an inherited disease.	○	○	○
Q11_5 A person with an altered (mutated) gene may be completely healthy.	○	○	○
Q11_6 All serious diseases are inherited.	○	○	○
Q11_7 Genes are instructions for making proteins, which help the body grow and work properly.	○	○	○
Q11_8 The child of a person with an inherited disease will always have the same disease.	○	○	○
Q11_9 A gene is a piece of DNA.	○	○	○
Q11_10 Altered (mutated) genes can cause disease.	○	○	○
Q11_11 Genes are inside of cells.	○	○	○
Q11_12 A chromosome contains many genes.	○	○	○
Q11_13 Genes determine traits such as height, eye color, and facial appearance.	○	○	○
Q11_14 A person has thousands of genes.	○	○	○
Q11_15 Identical twins have different sets of genes.	○	○	○
Q11_16 Humans have 20 pairs of chromosomes.	○	○	○

Q11_17 Parents pass both copies of each chromosome to their child.	o	o	o
Q11_18 A genetic test can tell you if you have a higher chance to develop a specific disease.	o	o	o

Q12_1. On a scale from 1 to 7 where 1 is not at all important, and 7 is very important, please mark how important it is to you to learn more about how your genes may affect your chance of getting cancer.

Not at all important			Somewhat important			Very important
○	○	○	○	○	○	○
1			4			7

Q12_2. …the health of any future children.

Not at all important			Somewhat important			Very important
○	○	○	○	○	○	○
1			4			7

Section 3:

For the next set of questions, we'd like to find out more about how you think about health and health information.

On a scale from 1 to 5 where 1 is strongly disagree, and 5 is strongly agree, how much do you agree or disagree with the following statements:

	Strongly disagree	Somewhat disagree	Neither agree nor disagree	Somewhat agree	Strongly agree
Q14_1 Living life in best possible health is very important to me	○ 1	○ 2	○ 3	○ 4	○ 5
Q14_2 Eating right, exercising, and taking preventive measures will keep me healthy for life	○ 1	○ 2	○ 3	○ 4	○ 5
Q14_3 My health depends on how well I take care of myself	○ 1	○ 2	○ 3	○ 4	○ 5
Q14_4 I actively try to prevent disease and illness	○ 1	○ 2	○ 3	○ 4	○ 5

Q14_5 I do everything I can to stay healthy	○ 1	○ 2	○ 3	○ 4	○ 5
Q14_6 I make a point to read and watch stories about health	○ 1	○ 2	○ 3	○ 4	○ 5
Q14_7 I really enjoy learning about health issues	○ 1	○ 2	○ 3	○ 4	○ 5
Q14_8 To be and stay healthy it's critical to be informed about health issues	○ 1	○ 2	○ 3	○ 4	○ 5
Q14_9) The amount of health information available today makes it easier for me to take care of my health	○ 1	○ 2	○ 3	○ 4	○ 5
Q14_10) When I take medicine, I try to get as much information as possible about its benefits and side effects	○ 1	○ 2	○ 3	○ 4	○ 5
Q14_11) I need to know about health issues so I can keep myself and my family healthy	○ 1	○ 2	○ 3	○ 4	○ 5
Q14_12) Before making a decision about my health, I find out everything I can about this issue	○ 1	○ 2	○ 3	○ 4	○ 5
Q14_13) It's important to me to be informed about health issues	○ 1	○ 2	○ 3	○ 4	○ 5

Q15) In the past 30 days, how often would you say you have looked for information about ways to stay healthy or to feel better?

- ☐ Very often
- ☐ Somewhat often
- ☐ Not very often
- ☐ Not at all

Q16. Compared to other people your gender, age, and race, how likely do you think you are to get each of the following types of cancer in your lifetime?

	A lot less likely	Somewhat less likely	About as likely	Somewhat more likely	A lot more likely
Q16_1) Breast cancer	o	o	o	o	o
Q16_2) Ovarian cancer	o	o	o	o	o
Q16_3) Colon cancer	o	o	o	o	o

On a scale from 1 to 7 where 1 is strongly disagree, and 7 is strongly agree, please mark how much you agree or disagree with the following statement:

Q17) The people who mean the most to me think I should learn more about ways I can keep myself healthy.

Strongly disagree			Neither agree nor disagree			Strongly agree
○	○	○	○	○	○	○
1			4			7

Q18) **On a scale from 1 to 7 where 1 is not at all motivated, and 7 is very motivated,** how motivated you would say you are to do what these people want you to do?

Not at all motivated			Somewhat motivated			Very motivated
○	○	○	○	○	○	○
1			4			7

On a scale from 1 to 5 where 1 is not at all characteristic of me, and 5 is entirely characteristic of me, please check the best answer for the following statements:

	Not at all characteristic of me				Entirely characteristic of me
Q19_1) Unforeseen events upset me greatly	○ 1	○ 2	○ 3	○ 4	○ 5
Q19_2) It frustrates me not having all the information I need.	○ 1	○ 2	○ 3	○ 4	○ 5
Q19_3) One should always look ahead so as to avoid surprises.	○ 1	○ 2	○ 3	○ 4	○ 5
Q19_4) A small, unforeseen event can spoil everything, even with the best of planning.	○ 1	○ 2	○ 3	○ 4	○ 5

Q19_5) I always want to know what the future has in store for me.	○ 1	○ 2	○ 3	○ 4	○ 5
Q19_6) I can't stand being taken by surprise.	○ 1	○ 2	○ 3	○ 4	○ 5
Q19_7) I should be able to organize everything in advance.	○ 1	○ 2	○ 3	○ 4	○ 5
Q19_8) Uncertainty keeps me from living a full life.	○ 1	○ 2	○ 3	○ 4	○ 5
Q19_9) When it's time to act, uncertainty paralyzes me.	○ 1	○ 2	○ 3	○ 4	○ 5
Q19_10) When I am uncertain I can't function very well.	○ 1	○ 2	○ 3	○ 4	○ 5
Q19_11) The smallest doubt can stop me from acting.	○ 1	○ 2	○ 3	○ 4	○ 5
Q19_12) I must get away from all uncertain situations.	○ 1	○ 2	○ 3	○ 4	○ 5

<u>Section 4:</u>

For the last set of questions, we'd like to ask more about you.

Q22) What is the highest level of school that you have completed?

- O Junior high or some High school
- O High school degree or GED
- O Some college
- O Associate degree
- O College degree
- O Graduate degree

Q24) What is your race? (Check all that apply)

- O White/Caucasian
- O African-American/Black
- O Asian
- O Pacific Islander/Native Hawaiian
- O Native American/Alaska Native
- O Other ___________________________

Q25) Do your parents or grandparents have any Ashkenazi (Eastern European) Jewish ancestry?

- O Yes
- O No
- O Not sure

Q26) What is your marital status?

- O Married
- O Living as married
- O Divorced
- O Separated
- O Never been married
- O Widowed

Q27) Do you have any biological children?

- □ Yes
- □ No

Q28) Are you planning to become pregnant in the next year?

- O Yes
- O No
- O Not sure

Q29) What is your zip code? ________

Q30) Think about your household's total income. About how much did your household receive in the last year?

- O Less than $25,000
- O $25,000 - $49,999
- O $50,000 - $74,999
- O $75,000 - $99,999
- O $100,000 or higher
- O Prefer not to answer

Q31) Do you currently have health insurance?

- O Yes, I have private insurance (for example, through my job, HMO)
- O Yes, I have public insurance (for example, Medicaid, Medicare)
- O No

Q32) Have you ever received genetic testing as part of your clinical care?

- □ Yes. {(Q33) if yes, what type_____________)
- □ No
- □ Don't know

Q34) Have you ever been diagnosed as having cancer?

- □ Yes. {(Q35) if yes, what type_____________)
- □ No

Q36) Have any of your family members ever had cancer?

- □ Yes. {(Q37) if yes, what type_____________)
- □ No

The next questions are about how you prefer to get information. For each of the following questions, please circle the number that best reflects your answer:

Q38) When reading the newspaper, how helpful do you find tables and graphs that are part of a story?

Not at all helpful					Extremely helpful
1	2	3	4	5	6

Q39) When people tell you the chance of something happening, do you prefer that they use words (e.g., "it rarely happens") or numbers (e.g., "there's a 1% chance")?

Always prefer words					Always prefer numbers
1	2	3	4	5	6

Q40) When you hear a weather forecast, do you prefer predictions using percentages (e.g., "there will be a 20% chance of rain today") or predictions using only words (e.g., "there is a small chance of rain today")?

Always prefer words					Always prefer percentages
1	2	3	4	5	6

Q41) How often do you find numerical information to be useful?

Never					Very often
1	2	3	4	5	6

For each of the following questions, please mark the number that best reflects how good you are at doing the following things:

Q42_1) How good are you at working with fractions?

Not at all good					Extremely good
1	2	3	4	5	6

Q42_2) How good are you at working with percentages?

Not at all good					Extremely good
1	2	3	4	5	6

Q42_3) How good are you at calculating a 15% tip?

Not at all good					Extremely good
1	2	3	4	5	6

Q42_4) How good are you at figuring out how much a shirt will cost if it is 25% off?

Not at all good					Extremely good
1	2	3	4	5	6

Q43) How often do you have someone (like a family member, friend, hospital/clinic worker or caregiver) help you read hospital materials?

None of the time	A little of the time	Some of the time	Most of the time	All of the time
1	2	3	4	5

Q44) How often do you have problems learning about your medical condition because of difficulty understanding written information?

None of the time	A little of the time	Some of the time	Most of the time	All of the time
1	2	3	4	5

Q45) How confident are you filling out medical forms by yourself?

Not at all	A little bit	Somewhat	Quite a bit	Extremely
1	2	3	4	5

That is the end of the questions. Do you have anything else that you would like to share with the research team?

Thank you again for agreeing to share this information with us.
